# Cardiac Function Remains Impaired Despite Reversible Cardiac Remodeling after Acute Experimental Viral Myocarditis

**DOI:** 10.1155/2017/6590609

**Published:** 2017-03-02

**Authors:** Peter Moritz Becher, Frauke Gotzhein, Karin Klingel, Felicitas Escher, Stefan Blankenberg, Dirk Westermann, Diana Lindner

**Affiliations:** ^1^Clinic for General and Interventional Cardiology, University Heart Center Hamburg, Hamburg, Germany; ^2^German Center for Cardiovascular Research (DZHK), Partner Site Hamburg/Kiel/Lübeck, Hamburg, Germany; ^3^Department of Molecular Pathology, Institute for Pathology, Eberhard-Karls-University Tübingen, Tübingen, Germany; ^4^German Center for Cardiovascular Research (DZHK), Partner Site Berlin, Germany; ^5^Department of Cardiology, Charité-Universitätsmedizin Berlin, Campus Virchow-Klinikum, Augustenburger Platz 1, 13353 Berlin, Germany

## Abstract

*Background*. Infection with Coxsackievirus B3 induces myocarditis. We aimed to compare the acute and chronic phases of viral myocarditis to identify the immediate effects of cardiac inflammation as well as the long-term effects after resolved inflammation on cardiac fibrosis and consequently on cardiac function.* Material and Methods*. We infected C57BL/6J mice with Coxsackievirus B3 and determined the hemodynamic function 7 as well as 28 days after infection. Subsequently, we analyzed viral burden and viral replication in the cardiac tissue as well as the expression of cytokines and matrix proteins. Furthermore, cardiac fibroblasts were infected with virus to investigate if viral infection alone induces profibrotic signaling.* Results*. Severe cardiac inflammation was determined and cardiac fibrosis was consistently colocalized with inflammation during the acute phase of myocarditis. Declined cardiac inflammation but no significantly improved hemodynamic function was observed 28 days after infection. Interestingly, cardiac fibrosis declined to basal levels as well. Both cardiac inflammation and fibrosis were reversible, whereas the hemodynamic function remains impaired after healed viral myocarditis in C57BL/6J mice.

## 1. Introduction

Myocarditis is defined as inflammation of the myocardium following myocardial injury [1–3]. It is one of the leading causes of heart failure (HF) in patients less than 40 years of age [4]. In most cases, the etiology of myocarditis is unknown. Nevertheless, infection with enteroviruses is thought to be one leading cause of the disease [3, 5], especially infection with cardiotropic Coxsackievirus B3 (CVB3) in young patients [6]. Acute viral myocarditis is described by focal cellular infiltrates with necrosis or fibrosis, primarily consisting of macrophages, CD4^+^ T cells, and CD8^+^ T cells with B cells, mast cells, dendritic cells, and natural killer cells [3, 6–12]. There exists evidence that especially B cells, T-helper cells, and macrophages are infected during the acute myocarditis suggesting indirect transport of virus particles into organs, which in turn leads to infection of resident cells [8, 9].

Increased gene expression of pro- and anti-inflammatory as well as antiviral cytokines could be observed in cardiac tissue of mice already 3 to 4 days after CVB3 infection [13–15]. However, inflammatory cytokines can be expressed on the one hand due to the infiltration of inflammatory cells and on the other hand due to activation of resident cardiac cells triggered by viral infection [10, 16–18]. Importantly, viral infection of resident cardiac cells (especially cardiomyocytes and fibroblasts) and of inflammatory cells is well documented [8, 9, 15, 16, 19–22].

The here described model of viral myocarditis is induced by a single intraperitoneal (i.p.) inoculation of C57BL/6J (B6) mice with heart-passaged CVB3 (Nancy strain) [7, 23]. Susceptibility to viral infections varies widely among individual inbred mouse strains. Acute viral myocarditis develops in susceptible (i.e., A.BY/SnJ) as well as resistant (i.e., C57BL/6J) mice from day 3 to 14 after viral infection [10, 17]. During the acute phase of myocarditis, viral genome is detected in the cardiac tissue of both susceptible and resistant strains of mice [7, 9, 13, 16, 17, 23–25]. Several immunocompetent mouse strains develop a chronic form of myocarditis that is associated with virus persistence, whereas resistant mouse strains eliminate the virus during acute myocarditis [7–9, 17, 26]. The differential susceptibility of diverse inbred mouse strains to CVB3-induced myocarditis was found to be associated with quantities of cytokine secretion, CVB3-neutralizing antibodies, and Toll-like receptor expression [10, 26]. However, chronic inflammatory cardiomyopathy and progress to dilated cardiomyopathy (DCM) behave differently between resistant and susceptible inbred strains of mice.

We aimed to investigate the long-term effects in view of the reversible and irreversible changes of the myocardium after resolved cardiac inflammation in the C57BL/6J mouse strain that overcome the CVB3 infection during the acute phase. Extensive degradation and adverse cardiac remodeling of the myocardium, largely dependent on the inflammatory immune response, may lead to cardiac slippage with dilation and left ventricular dysfunction. Here, we provide an analysis of hemodynamic function, status in cardiac inflammation and myocardial fibrosis, and their pathophysiological interplay during the acute and chronic disease stadium of experimental myocarditis.

## 2. Material and Methods

### 2.1. Study Design

Male C57BL/6J mice were used at an age of 6 to 10 weeks. Mice were infected intraperitoneally with 5 × 10^5^ plaque forming units (pfu) of Coxsackievirus B3 (CVB3) (nancy strain) diluted in PBS, whereas sham infections were performed using PBS as control subjects (*n* = 6–9 mice per group). Seven days as well as 28 days later CVB3-infected C57BL/6J mice were hemodynamically characterized and compared to noninfected healthy control mice (PBS treated). This investigation conforms to the Guide for the Care and Use of Laboratory Animals published by the US NIH (NIH Publication number 85-23, revised 1996).

### 2.2. Hemodynamic Measurements

For hemodynamic measurements a microconductance catheter (1.2F) system in open-chest animals was used as described previously [27, 28]. Animals were anesthetized using urethane (0.8–1.2 g/kg) and 0.05 mg/kg buprenorphine (i.p.) intubated and artificially ventilated. A 1.2F-microconductance pressure catheter (SciSense, Ontario, Canada) was positioned in the left ventricle via the apex for continuous registration of pressure-volume loops. Global function was analyzed by heart rate (bpm), cardiac output (mL/min), stroke volume (*μ*L), and stroke work (*μ*L×mmHg). To investigate the systolic function end-systolic pressure (*P*_es_ in mmHg), left ventricular contractility (d*P*/d*t*_max_ in mmHg/s) and end-systolic volume (*V*_es_ in *μ*L) were assessed. Diastolic function was determined by end-diastolic pressure (*P*_ed_ in mmHg), left ventricular relaxation (d*P*/d*t*_min_ in mmHg/s), left ventricular relaxation time (Tau in ms), and end-diastolic volume (*V*_ed_ in *μ*L).

Hearts of sacrificed animals were removed and immediately frozen in liquid nitrogen and stored at −80°C for further histological or molecular analyses.

### 2.3. Immunohistochemistry and Histological Measurements

Histological analyses were carried out on 5 *μ*m thick frozen cross sections of the murine hearts. To analyze the cardiac inflammation in general, cross sections were stained using hematoxylin and eosin to visualize inflammatory parts of cardiac tissue.

Further, sections were stained with antibodies directed against CD11b (rat anti-CD11b; Pharmingen), CD68 (rat anti-CD68, abcam), or CD80 (rat anti-CD80, Pharmingen). As secondary antibody a biotinylated rabbit anti-rat antibody was used. The specific antigen was visualized using biotin-streptavidin-peroxidase method (Vectorlabs). Furthermore, primary antibodies detecting collagen-I (rabbit anti-Col-I; Chemicon) and collagen-III (rabbit anti-Col-III; Calbiochem) were used and visualized by Envision peroxidase technique (Dako). Finally sections were counterstained with hemalum.

Documentation of the histological staining was performed on Keyence BZ-9000 microscope. Therefore, multiple images of one section were captured. Images were merged with the image stitching function, resulting in an overview image of the LV cross section. For each animal, six sections consisting of three different layers of the heart, performed in duplicate, were acquired. Area fraction of the antibody stained part was calculated using BZ-II Analyzer software. Positive cells per mm^2^ tissue were determined using an existing algorithm in ImageJ. Therefore, pictures were converted to 8-bit images and a universal threshold was set, including only positively stained areas. Outliers, with a minimum radius of 15 pixels, were removed to avoid false positive results and binary images were created. Ultimate points from the Euclidean distance map of each image were counted and assumed to present infiltrated immune cells.

GIMP 2 software was used to remove surrounding cell debris or Tissue-Tek leftovers, which should be excluded for quantification.

### 2.4. Measurement of Protein Expression

Proteins were isolated from left ventricles of healthy C57BL/6J and CVB3-infected animals using cell lysis buffer (Cell Signaling, UK) with added protease and phosphatase inhibitors. Tissue was disrupted using pellet pestle followed by vigorous shaking at 800 rpm and 4°C for 10 minutes. In order to analyze protein expression in tissue a multiplex Bioplex chemokine Assay (Bio-Rad, Germany) was used. Using a handheld magnetic washer (Bio-Rad, Germany) and flat bottom plates, the procedure was performed in accordance with the recommended protocol. Diluted magnetic beads were applied on the assay plate and washed. Samples as well as a protein standard and water as a negative control were added. Each measurement was performed in duplicate. Then, the assay plate was incubated for 1 h at room temperature and 850 rpm followed by 1 h incubation with appropriately diluted detection antibodies (Bio-Rad, Germany). Subsequently, detection antibodies were visualized using streptavidin-PE (Bio-Rad, Germany) and analysis was performed with Bioplex 200 System (Bio-Rad, Germany). A minimum of 50 beads per well was counted and gated based on the bead color. Chemokine concentrations were quantified via standard concentration curves and evaluated by using Bioplex Manager software (Bio-Rad, Germany).

In order to analyze relative levels of cytokines in the serum a Mouse Cytokine Array Panel A (R&D Systems, USA) was performed corresponding to the manufacturer's instructions. In brief, nitrocellulose membranes carrying capture spotted antibodies against 40 common cytokines (in duplicate) were blocked for 1 hour at room temperature. Subsequently, serum was diluted 1 : 15 in the supplied buffers and reconstituted Mouse Cytokine Array Panel A Detection Antibody Cocktail was added to the solutions. After 1 h of incubation the serum antibody mixture was applied on the membranes and was incubate overnight at 4°C. After several washing steps, bound secondary antibodies were detected with streptavidin-HRP and SuperSignal Femto substrate (Thermo Fisher Scientific, USA). Chemiluminescence was visualized using Fusion Solo S imaging system (Vilber, Germany). Cytokine levels were quantified as pixel densities using ImageJ dot blot analyzer tool. Average signals of duplicates were calculated for each cytokine after subtracting values of negative control dots. All values were normalized to the reference spots of healthy control animals.

### 2.5. RNA Isolation from Tissue Sections

Total RNA was isolated from frozen tissue sections using Trizol reagent. Disruption of the tissue occurred during 10 min of vigorous shaking, followed by extraction of RNA by adding chlorophorm. After mixing and centrifugation, the aqueous phase containing the RNA was collected. Isopropanol was added and the samples were centrifuged for 15 min at 4°C at high speed to precipitate the RNA. The obtained RNA pellet was further purified using RNeasy Mini Kit (Qiagen, Germany) following the manufacturer's instructions. Determination of the nucleic acid concentration was performed by measuring the absorbance at 260 nm using a Nanodrop 2000c spectrophotometer. RNA was stored at −80°C for further analyses.

### 2.6. Cell Culture

The left ventricles from 12-week-old male C57BL/6J mice were used to obtain primary murine cardiac fibroblasts as described previously [29, 30]. The atrium and the right ventricle were removed and discarded using a fine surgical scissor purchased from Fine Science Tools (FST, Germany). To isolate the cardiac fibroblasts, left ventricles were cut into small pieces and digested in 0.1 mg/mL liberase dissolved in HBSS at 37°C for 10 min subsequently repeated six times. Afterwards, isolated cells were transferred over a cell strainer to remove remaining tissue pieces. Murine cells were centrifuged and for cultivation suspended in Dulbecco's modified eagle medium (DMEM) containing 20% fetal calf serum, 100 U/mL penicillin and 100 *μ*g/mL streptomycin (Biochrom, Germany). To subculture confluent fibroblasts, cells were detached using Trypsin/EDTA for 3 min at 37°C. Characterization of the cells as fibroblasts was performed by positive immunofluorescence staining against collagen-I and negative staining for the myocyte and endothelial marker desmin and CD31, respectively, as described in our previous study [29]. Cell culture experiments were carried out in a humidified atmosphere with 5% CO_2_ and 95% air.

For viral infection cardiac fibroblasts were seeded out into 24-well plates and grown to confluence before starving in DMEM containing 0.5% FCS, 100 U/mL penicillin, and 100 *μ*g/mL streptomycin overnight. To calculate the multiplicity of infection (MOI) one well was used to determine cell numbers. Viral infection was performed with Coxsackievirus B3 (nancy strain) diluted to 0.5 MOI in DMEM without any supplements for 60 min. To completely remove remaining virus particles the medium containing CVB3 had to be replaced. Cells were washed twice using PBS and incubated in starving medium for further 23 hours before RNA extraction was performed. All control cells underwent equal treatment in the absence of CVB3.

For TGF-*β* stimulation, cardiac fibroblasts were seeded into 24-well plates, cultured until they reached confluence, and then starved in DMEM containing 0.5% FCS, 100 U/mL penicillin, and 100 *μ*g/mL streptomycin overnight. TGF-*β* stimulation was performed using a final concentration of 5 ng/mL TGF-*β* in starving medium. Cardiac fibroblasts were incubated in the presence or absence of TGF-*β* for 24 hours and stopped by cell lysis.

Finally cells were lysed in RLT buffer (RNeasy Kit, Qiagen, Germany) containing 1%  *β*-mercaptoethanol. To isolate total RNA from cells RNeasy Mini Kit (Qiagen, Germany) was used according to the manufacturer's protocol.

### 2.7. Reverse Transcription and Relative Gene Expression Analysis

Reverse transcription of RNA was carried out using the High Capacity Kit (Life Technologies, Germany). Therefore, 250 ng total RNA isolated from murine cells or 1 *μ*g total RNA isolated from cardiac tissue was reversely transcribed for 2 h at 37°C followed by an inactivation step for 5 min at 85°C. The resulting cDNA was diluted to a final working concentration of 1.25 ng/*μ*L for cell culture samples and 10 ng/*μ*L for tissue samples.

Relative quantification of RNA expression was performed with a 7900 TaqMan system (Applied Biosystems, Germany). To determine RNA expression levels of various genes, real-time PCR was carried out using 5 *μ*L of gene expression master mix (Life technologies, Germany) and 0.5 *μ*L of the gene expression assay for* Mcp-1 (Ccl2)* (Mm99999056_m1),* Rantes (Ccl5)* (Mm01302428_m1),* Mcp-3 (Ccl7)* (Mm00443113_m1),* Ip-10 (Cxcl10)* (Mm99999072_m1),* Cxcl13* (Mm00444534_m1),* Il-6* (Mm00446190_m1),* Il-23a* (Mm00518984_m1),* Tnf-α* (Mm00443258_m1),* Cd3* (Mm01179194_m1),* Cd4* (Mm00442754_m1),* Cd8* (Mm01182107_g1),* Cd19* (Mm00515420_m1),* Col1a1* (Mm01302043_g1),* Col3a1* (Mm00802331_m1),* Ctgf* (Mm00515790_g1),* Tgf-β1* (Mm00441724_m1),* Timp-1* (Mm00441818_m1), or* Mmp13* (Mm01168712_m1) purchased from Life Technologies. The assay for gene expression consists of gene specific forward and reverse primers and the FAM-labelled probe. As template, 1 *μ*L cDNA was used in a final volume of 10 *μ*L, each performed in duplicate. As an endogenous control, the gene expression of 18S (Hs99999901_s1) or* Cdkn1b* (Mm00438167_g1) was assessed. The absolute gene expression data were normalized to the housekeeping gene by using the formula 2^−ΔCt^ and plotted as *x*-fold to 18S or* Cdkn1b*, whereas the relative gene expression data were further normalized using the formula 2^−ΔΔCt^ and plotted as *x*-fold to untreated control as described previously [31].

### 2.8. CVB3 Strand-Specific RT-PCR and Copy Number Calculation

The viral RNA genome (+) strand was reverse-transcribed using CVB3_antisense primer (5′-ATTGTCACCATAAGCAGCCA-3′) whereas the intermediate RNA (−) strand of CVB3 was reverse-transcribed using CVB3_sense primer (5′-CCCTGAATGCGGCTAATCC-3′) in order to determine the strand-specific copy numbers. A concentration of 50 ng/*μ*L total RNA from cardiac tissue was transcribed with 30 ng/*μ*L of the CVB3 strand-specific primer in a final volume of 5 *μ*L using the High Capacity Kit (Life Technologies, Germany) as described above followed by an inactivation step at 85°C for 1 hour. The obtained cDNA was further diluted to a final concentration of 10 ng/*μ*L and used to determine the strand-specific copy numbers of CVB3. To verify the inactivation of the reverse transcriptase, total RNA was incubated with the enzyme in absence of strand-specific primers during transcription followed by the inactivation step at 85°C.

To determine the copy numbers of the (+) or (−) strand of CVB3, gene expression analysis was performed as described above replacing the gene expression assays by the forward primer CVB3_sense (5′-CCCTGAATGCGGCTAATCC-3′), the reverse primer CVB3_antisense (5′-ATTGTCACCATAAGCAGCCA-3′) in a final concentration of 15 ng/*μ*L, and the FAM-labelled CVB3-MGB-probe (5′-TGCAGCGGAACCG-3′) in a final concentration of 0.25 pmol/*μ*L. A standard curve was created by plotting a serial dilution of a plasmid containing the amplified CVB3 sequence. This standard curve was used to determine the copy numbers of CVB3.

### 2.9. Statistical Analysis

All statistical analyses were performed using Graph Pad Prism 6 software (GraphPad Software, La Jolla, CA). Statistical comparison of two groups was performed using the Mann–Whitney *U* test with *P* values < 0.05 considered statistically significant. More than two groups were compared using Kruskal-Wallis-test followed by Dunn's posttest with *P* values < 0.05 considered statistically significant.

## 3. Results

### 3.1. Impaired Hemodynamic Function 7 and 28 Days after CVB3-Induced Viral Myocarditis

C57BL/6J mice were intraperitoneally infected with 5 × 10^5^ pfu of CVB3 (Nancy strain) and hemodynamically characterized 7 and 28 days after viral infection. As shown in Table 1, CVB3-infected mice did significantly lose weight within 7 days, which remained at a similar level in the chronic phase of the disease 28 days after infection. Furthermore, the heart weight was significantly reduced 7 as well as 28 days after infection compared to healthy control animals. Using the hemodynamic parameters heart rate, cardiac output, stroke volume, and stroke work, the global LV function was characterized and revealed an impaired global LV function with significantly decreased heart rate and stroke volume resulting in reduced cardiac output and stroke work compared to healthy controls.

In addition, systolic and diastolic LV function of CVB3-infected mice were significantly impaired in comparison to noninfected healthy control mice 7 as well as 28 days after infection. Comparing the LV function of mice within the acute phase of myocarditis (7 days p.i.) with those in the chronic phase of the disease (28 days p.i.) no significantly different parameters were determined.

However, in turn a mild improvement in systolic and diastolic function 28 days after CVB3 infection (chronic disease stadium) was detected. Compared to healthy controls a significantly reduced end-systolic pressure *P*_es_ was detected 7 days after infection (−43%) but 28 days after infection a slightly and not longer significantly decreased *P*_es_ was measured (−17%). Similar findings are also shown for the end-diastolic pressure *P*_ed_ which was significantly increased after 7 days (+76%). After 28 days *P*_ed_ was decreased compared to 7 days but still increased compared to control animals (+22%).

### 3.2. Severe Cardiac Inflammation and Virus Replication during Acute Viral Myocarditis Was Clearly Reduced in the Chronic Phase

**Figure 1:**
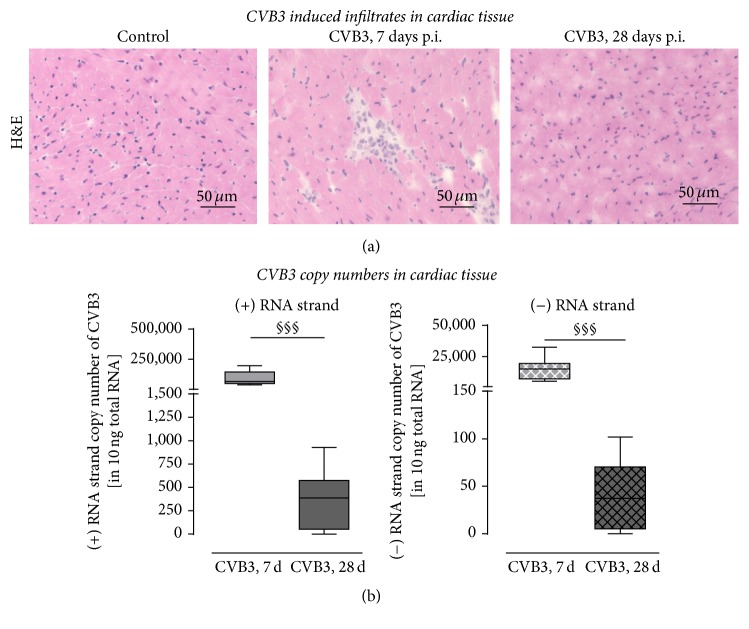
Cardiac inflammation and viral load in C57BL/6J mice 7 and 28 days after CVB3 infection. (a) Hematoxylin/eosin staining of cardiac tissue revealed higher numbers of invaded inflammatory cells 7 days after infection compared to control tissue. However, 28 days after infection inflammatory cells are hardly detectable and thus remarkably reduced compared to 7 days after infection. (b) Viral load in cardiac tissue of CVB3-infected C57BL/6J mice after 7 and 28 days. To determine the amount of progeny positive-strand RNA or the intermediate negative-strand RNA of CVB3, cDNA synthesis was performed with (+) or (−) strand-specific primers followed by quantification. More (+) RNA than (−) RNA was detected in cardiac tissue 7 as well as 28 days after infection. The amount of both strands was clearly reduced between 7 and 28 days demonstrating the elimination of CVB3 virus particles as well as the elimination of infected cells. However, the (+) RNA strand was detected in a higher extent compared to the (−) RNA strand; ^§^significantly different compared to 7 days p.i.; ^§§§^*P* < 0.001.

To assess the cardiac inflammation 7 as well as 28 days after peritoneal CVB3 infection, frozen tissue cross sections were stained using hematoxylin and eosin to visualize infiltration of inflammatory cells. While invaded cells were clearly visible 7 days p.i. at the foci of inflammation, no inflammatory cells could be seen 28 days after infection (Figure 1(a)).

Comparable results were determined for the viral load 7 and 28 days after CVB3 infection. In order to investigate the replication of CVB3 7 and 28 days after intraperitoneal injection, the viral copy numbers were assessed by strand-specific quantitative RT-PCR (Figure 1(b)). Therefore, total RNA was isolated from cardiac tissue of C57BL/6J mice infected with CVB3 and noninfected animals as healthy controls. Reverse transcription was performed utilizing a CVB3 (+) or (−) RNA strand-specific primer followed by TaqMan analysis to determine the CVB3 copy numbers. These analyses revealed high copy numbers of the viral genome, indicated as the (+) RNA strand, in the cardiac tissue 7 days after infection, whereas the copy numbers of the intermediate (−) RNA strand were remarkably lower expressed. The copy numbers of both viral RNA strands were remarkably reduced to less than 1000 copies of (+) RNA strand in 10 *μ*g total RNA and less than 100 copies of the (−) RNA strand in 10 *μ*g total RNA. Interestingly, in two out of seven animals no viral genome could be detected in the cardiac tissue 28 days after infection.

**Figure 2:**
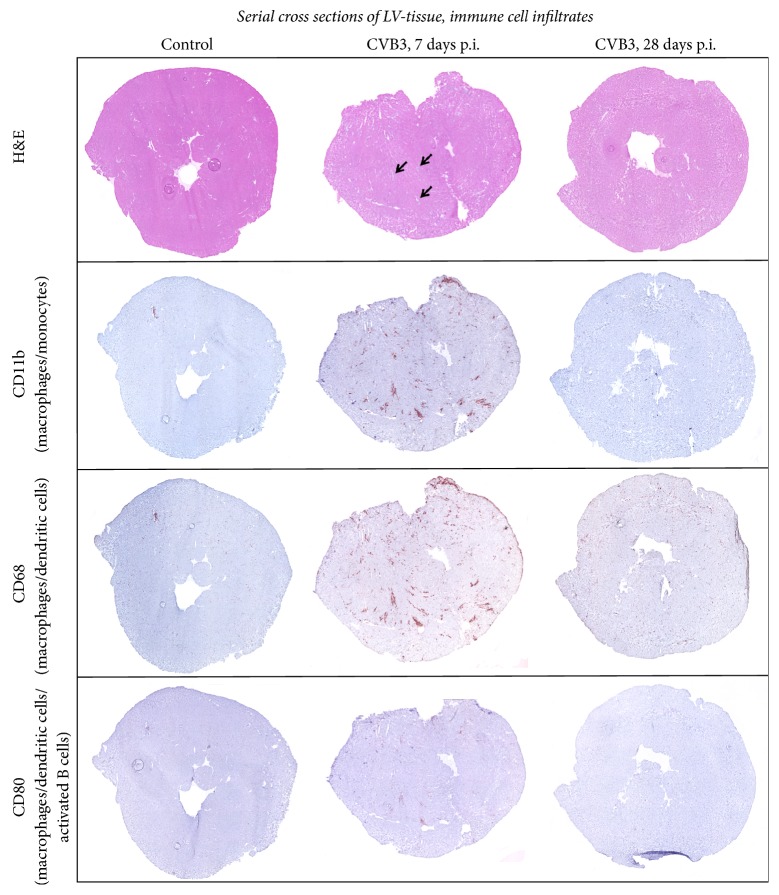
Immune cell infiltration: serial cross section of LVs from C57BL/6J mice without viral infection or 7 as well as 28 days after intraperitoneal CVB3 infection. Tissue sections were stained for various markers of inflammatory cells. Hematoxylin/eosin staining showed higher numbers of invaded cells to foci of inflammation 7 days after infection (indicated by arrows) compared to 28 days after infection. Increased numbers of CD80^+^, CD11b^+^, and CD68^+^ cells are detected within the identified foci of inflammation 7 days after infection.

**Figure 3:**
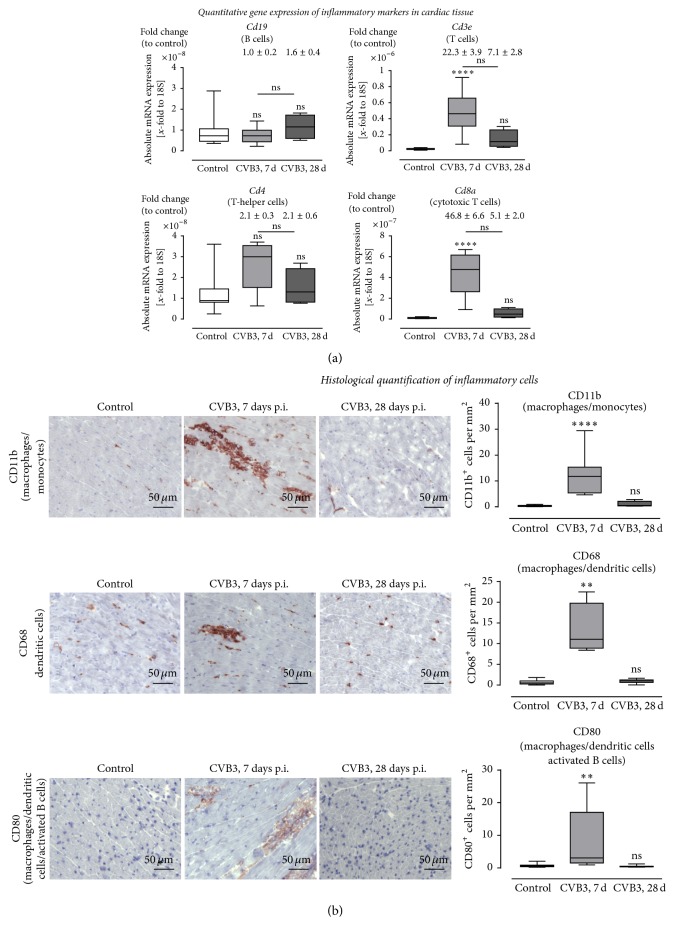
Immune cell infiltration: gene expression and histological quantification of specific marker for various inflammatory cells in cardiac tissue of CVB3-infected mice 7 and 28 days after infection. (a) Gene expression analysis of specific marker for various immune cells. The gene expression of* Cd3e*, as a marker for T cells in general, was highly increased 7 days after infection. Subsequent analysis of* Cd4* and* Cd8a* expression revealed that CD8^+^ cytotoxic T cell represents the predominant T cell population within the cardiac tissue during the acute phase of viral myocarditis. Furthermore,* Cd19* expression, as a marker for B cells, was not increased 7 and 28 days after infection. (b) Detailed images of murine cardiac tissue stained for different cell surface markers of immune cells. Immune cells were quantified and plotted as positive cells per mm^2^. Tissue sections stained for CD11b^+^, CD68^+^, and CD80^+^ cells revealed significantly increased numbers 7 days after CVB3 infection compared to healthy controls. 28 days after infection no increased inflammation was detectable. The white bar represents healthy C57BL/6J mice, and grey bars represent mice infected with CVB3 for 7 days (light grey) or for 28 days (dark-grey). Data are presented as box plots; ^*∗*^significantly different compared to noninfected mice (control); ^*∗∗*^*P* < 0.01; ^*∗∗∗∗*^*P* < 0.0001; ^ns^not  significant.

We further aimed to determine the differences of inflammation in cardiac tissue 7 and 28 days p.i., respectively. Frozen cross sections of murine left ventricles were stained with hematoxylin and eosin to visualize the foci of inflammation (indicated by arrows in Figure 2) as well as with antibodies directed against specific immune cell marker such as CD11b, CD68, and CD80. We performed the staining of serial cross sections and documented the immunohistological stainings as overview images (Figure 2) and as detailed images (Figure 3(b)). To evaluate the immune response detected within the cardiac tissue, we determined specific T cell and B cell marker as well as marker related to the innate immune system. In Figure 3 it is clearly shown that T cells, especially cytotoxic T cells, and cells of the innate immune system mainly represent the invaded inflammatory cells 7 days after CVB3 infection. The gene expression of the T-helper cell specific* Cd4* marker was slightly but not significantly increased, whereas the cytotoxic T cell specific marker* Cd8* was 47-fold increased 7 days after infection and 5-fold increased after 28 days after infection. The B cell specific marker* Cd19* was not increased during the acute or the chronic phase. Furthermore, the immunohistological stainings were quantified and presented as box plots in Figure 3(b). The cardiac tissue of infected mice at the acute state of myocarditis (7 days p.i.) revealed significantly increased numbers of CD11b^+^, CD68^+^, and CD80^+^ cells, whereas no increased inflammation was detected 28 days after CVB3 infection. Seven days after CVB3 infection CD11b^+^ cells were 33-fold increased, CD68^+^ cells were 19-fold increased, and CD80^+^ cells were 12-fold increased as shown in Figure 3(b). Cross sections of CVB3-infected C57BL/6J mice 28 days p.i. indicated no significant differences compared to control mice for all investigated immune cell markers.

### 3.3. Increased Expression of Proinflammatory Cytokines 7 Days after CVB3 Infection Declined to Basal Levels after 28 Days

**Figure 4:**
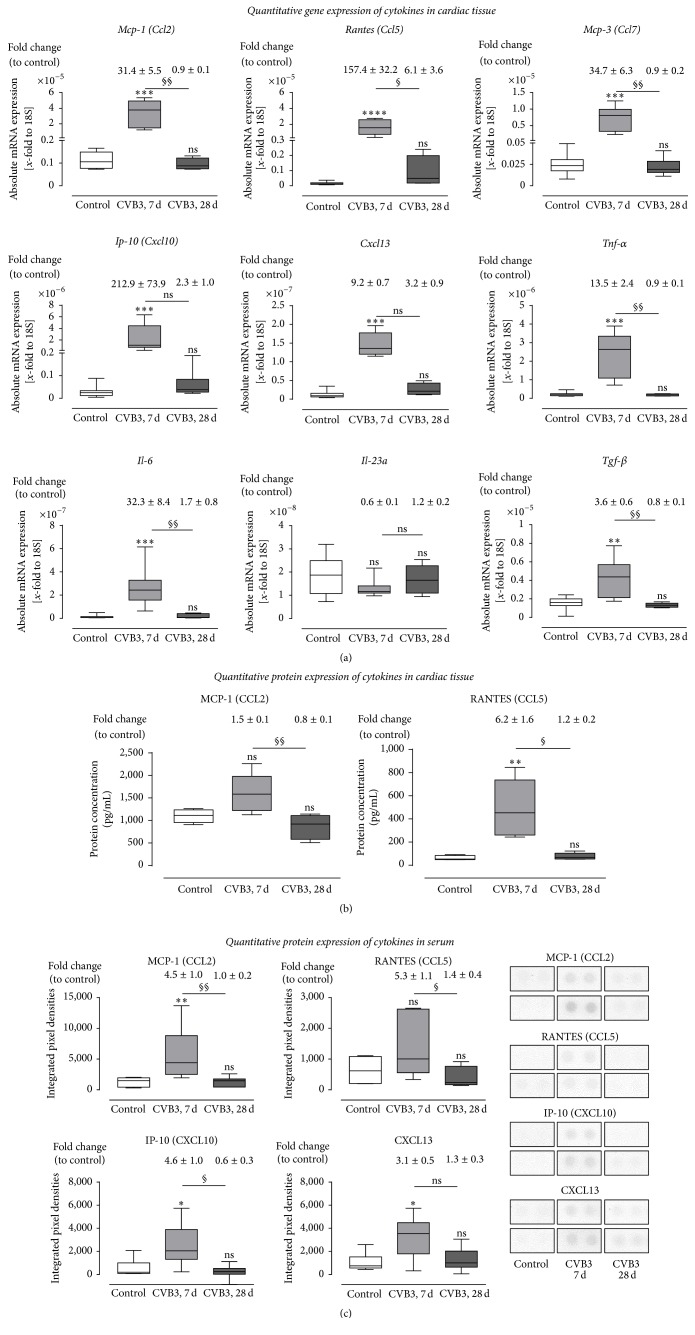
Cytokine expression in cardiac tissue and serum of CVB3-infected C57BL/6J mice 7 and 28 days after infection. (a) Cardiac tissue of CVB3-infected C57BL/6J mice was used for TaqMan based gene expression analysis of various chemotactic chemokines and cytokines. The gene expression of chemokines* (Ccl2*,* Ccl5*,* Ccl7*,* Cxcl10,* and* Cxcl13)* as well as the expression of* Il-6* and* Tnf-α* was highly increased 7 days p.i. compared to healthy controls and returned to basal levels 28 days after infection. Gene expression of* Il-23a* expression was not altered in cardiac tissue after CVB3 infection. Data are presented as absolute mRNA expression (*x*-fold to the house keeping gene 18S) in box plots as well as in fold change to control animals as mean ± SEM above the corresponding bar using the formula 2^−ΔΔCt^. (b) Protein expression in cardiac tissue was determined using Bioplex. The protein expression of MCP-1 and RANTES was increased 7 days after infection and dropped down 28 days after infection. (c) Protein expression in serum was detected using protein profiler arrays. The chemokines MCP-1, RANTES, IP-10, and CXCL13 were increased in serum samples of CVB3 infected mice 7 days after infection. Expression levels from noninfected control mice are shown as white bars, from CVB3-infected animals 7 days p.i. as light grey bars and from CVB3-infected animals 28 days p.i. as dark-grey bars; ^*∗*^significantly different compared to noninfected mice (control); ^*∗*^*P* < 0.5; ^*∗∗*^*P* < 0.01; ^*∗∗∗*^*P* < 0.001; ^*∗∗∗∗*^*P* < 0.0001; ^§^significantly different compared to 7 days p.i.; ^§^*P* < 0.05; ^§§^*P* < 0.01; ^ns^not  significant.

Furthermore, gene expression and protein expression analyses of cardiac tissue from CVB3-infected C57BL/6J mice were performed to prove cardiac inflammation in response to CVB3 infection. This was achieved by quantification of gene expression concerning various cytokines 7 and 28 days after viral infection compared to healthy controls. In Figure 4(a), absolute mRNA levels were plotted and relative gene expression levels compared to the healthy control mice are presented above the corresponding box plot. The chemokines* Mcp-1 (Ccl2)*,* Rantes (Ccl5)*,* Mcp-3 (Ccl7)*,* Ip-10 (Cxcl10),* and* Cxcl13* and the proinflammatory cytokines* Il-6* and* Tnf-α* showed significantly elevated gene expression levels in C57BL/6J mice 7 days after infection, whereas no significant differences were observed 28 days after infection.

Furthermore, as estimated, no expression of the antiviral cytokines* Ifn*-*β*1 and* Ifn*-*γ* could be detected in healthy controls, but they were highly expressed in CVB3-infected animals, 7 days after infection (data not shown). As further shown in Figure 4(a), the gene expression of the profibrotic growth factor* Tgf-β* was significantly increased 7 days after infection.

However, after 28 days a significant decrease of proinflammatory cytokines was determined in comparison to 7 days after infection, whereas the mRNA expression level of* Il-23a* was not regulated regarding CVB3 infection at any time point.

Furthermore, quantitative protein expression of cytokines in the cardiac tissue and in serum was determined and revealed comparable results as reported from the gene expression analysis. In Figure 4(b), protein expression of cardiac tissue was measured. MCP-1 (CCL2) as well as RANTES (CCL5) were elevated in cardiac tissue 7 days after infection and significantly declined in the chronic phase. Similar results are shown in serum samples (Figure 4(c)). MCP-1 (CCL2), RANTES (CCL5), IP-10 (CXCL10), and CXCL13 were elevated during the acute phase and decreased during the chronic phase.

### 3.4. Reversible Cardiac Fibrosis after Viral Myocarditis

Next, we studied the cardiac remodeling processes during viral myocarditis. Immunohistochemical stainings of collagen type I and collagen type III on frozen left ventricular cross sections from CVB3-infected and healthy mice were performed and quantified.

**Figure 5:**
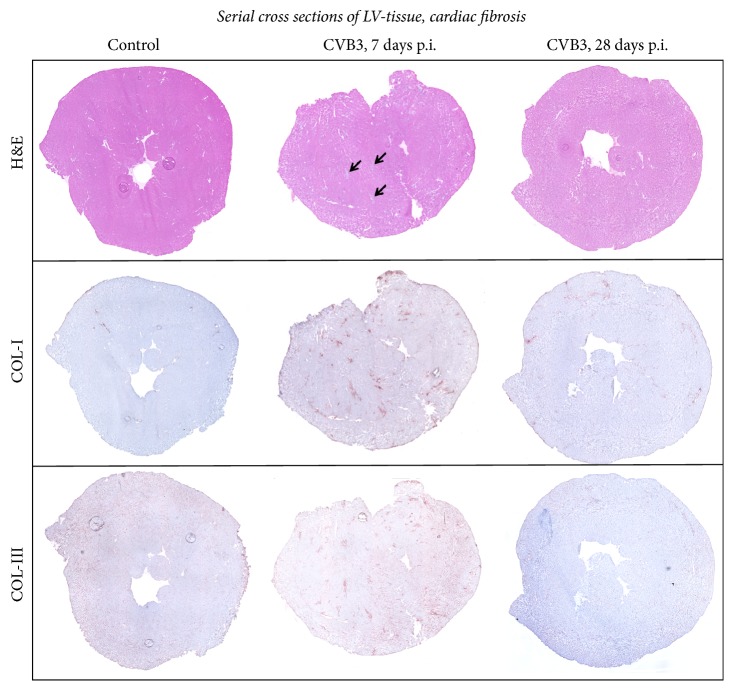
Remodeling: serial cross section of LVs from C57BL/6J mice without viral infection or 7 as well as 28 days after intraperitoneal CVB3 infection. Cardiac tissue of healthy or CVB3-infected C57BL/6J mice were stained for COL-I and COL-III. Hematoxylin/eosin staining revealed infiltrated cells. The foci of inflammation are clearly shown 7 days after infection indicated by arrows. Histological staining of COL-I showed a clear increase in fibrosis after CVB3 infection within the foci of inflammation. Immunohistochemistry of COL-III reveals no distinct effect of CVB3 infection.

**Figure 6:**
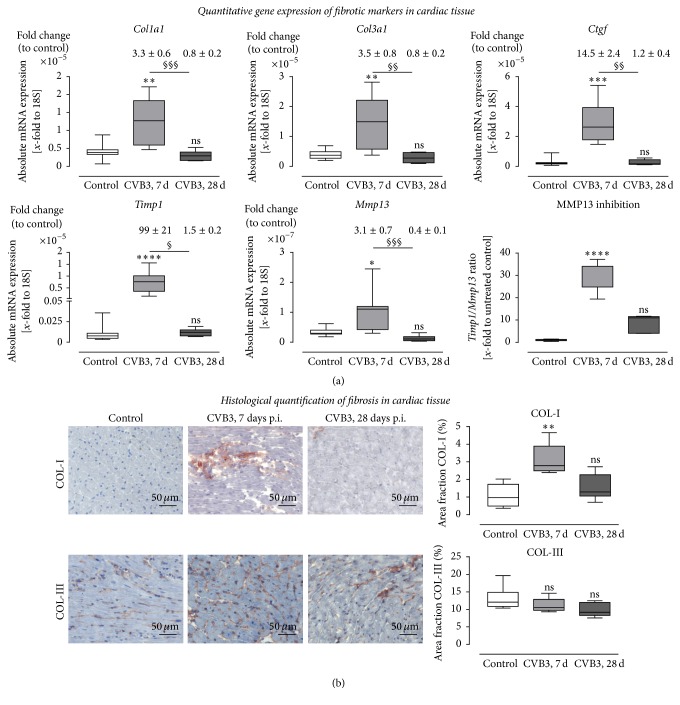
Expression analysis of remodeling processes in cardiac tissue of CVB3-infected C57BL/6J mice 7 and 28 days after infection. (a) Gene expression analysis in cardiac tissue of healthy (white bar) and CVB3-infected C57BL/6J mice 7 days p.i. (light grey bar) as well as 28 days p.i. (dark-grey bar) was determined using TaqMan analysis. The mRNA expression of profibrotic genes* Col1a1*,* Col3a1,* and* Ctgf* was increased in mice 7 days after CVB3 infection and dropped down to normal expression levels 28 days after infection. Furthermore, expression of genes involved in the regulation of ECM degradation was increased within 7 days after infection.* Timp1*, the endogenous inhibitors for MMPs, as well as the collagenase* Mmp13* was significantly upregulated in cardiac tissue 7 days after infection. Since the* Timp1* expression raised more than the* Mmp13* expression the ratio* Timp1*/*Mmp13* revealed an increased* Mmp13* inhibition. Data are presented as absolute mRNA expression (*x*-fold to the house keeping gene 18S) in box plots as well as in fold change to control animals as mean ± SEM above the corresponding bar using the formula 2^−ΔΔCt^. (b) Detailed histological stainings were performed to quantify cardiac remodeling and plotted as area fraction of healthy (white bar) and infected C57BL/6J mice 7 days p.i. (light grey bar) and 28 days p.i. (dark-grey bar). Tissue sections were stained for COL-I as well as COL-III. COL-I staining of cardiac tissue yielded in a significant increase of fibrosis in CVB3-infected mice 7 days after infection compared to healthy controls. Analyses of COL-III did not show significant changes in cardiac tissue after CVB3 infection; ^*∗*^significantly different compared to noninfected mice (control); ^*∗*^*P* < 0.05; ^*∗∗*^*P* < 0.01; ^*∗∗∗*^*P* < 0.001; ^*∗∗∗∗*^*P* < 0.0001; ^§^significantly different compared to 7 days p.i.; ^§^*P* < 0.05; ^§§^*P* < 0.01; ^§§§^*P* < 0.001;  ^ns^not  significant.

We performed stainings on serial cross sections and documented this as overview images (Figure 5) and as detailed images (Figure 6(b)). In Figure 5, we included again the hematoxylin and eosin staining and indicated the foci of inflammation by arrows. It is demonstrated in the overview images of serial cross sections that cardiac fibrosis is clearly colocalized with cardiac inflammation. Staining for collagen-I (COL-I) as well as collagen-III (COL-III) is shown for healthy controls and for mice infected with CVB3 7 or 28 days prior to investigations. Collagen type I protein expression was clearly elevated (3.4-fold) 7 days after CVB3 infection and interestingly declined to basal levels after 28 days (Figures 5 and 6(b)). The quantitative analyses are plotted as area fractions in Figure 6(b). Collagen type III showed no obvious alteration in response to CVB3 infection.

Furthermore, we have analyzed the gene expression level of genes involved in remodeling processes. Therefore, in Figure 6(a) absolute mRNA levels were plotted and above the corresponding box plot the relative gene expression levels are presented compared to healthy control mice. Gene expression levels of* Col1a1*,* Col3a1,* and* Ctgf* were determined in cardiac tissue 7 days and 28 days after CVB3 infection and compared to healthy controls. All three profibrotic genes were significantly upregulated in the cardiac tissue of infected animals 7 days after infection. 28 days after infection the expression declined to similar levels as described for healthy tissue. None of the investigated profibrotic genes was significantly upregulated 28 days after infection. Comparing the expression levels of cardiac tissue 7 and 28 days after infection, significant differences for all four genes were observed. Moreover, we evaluated the gene expression levels of the matrix-degrading enzyme* Mmp13* and the counterparts, the endogenous tissue inhibitor of metalloproteinases (*Timp1*), and* Timp1/Mmp13* ratio to illustrate the inhibition of matrix degradation leading to fibrosis. As shown in Figure 6(a), the expression level of* Timp1* as well as* Mmp13* was upregulated in cardiac tissue 7 days after CVB3 infection and declined to basal expression 28 days after infection. Since the expression level of* Timp1* was increased nearly 100-fold, the* Timp1*/*Mmp13* ratio was highly increased which results in matrix metalloproteinase inhibition and finally in fibrosis.

### 3.5. No Remodeling Processes with Virus Only: Cardiac Inflammation Contributes to Cardiac Remodeling

It was recently described that activated inflammatory cells increase the expression of the profibrotic growth factor TGF-*β* [30, 32]. Since cardiac fibroblasts are one major source of matrix proteins, we further examined matrix remodeling progression of cardiac fibroblasts during viral infection. 24 hours after infection of cardiac fibroblasts with 0.5 MOI of CVB3, copy numbers of (7.8 ± 2.8) × 10^6^/ng total RNA were detected (data not shown). Furthermore, those results were compared to TGF-*β* stimulated cardiac fibroblasts as a profibrotic stimulus.

**Figure 7:**
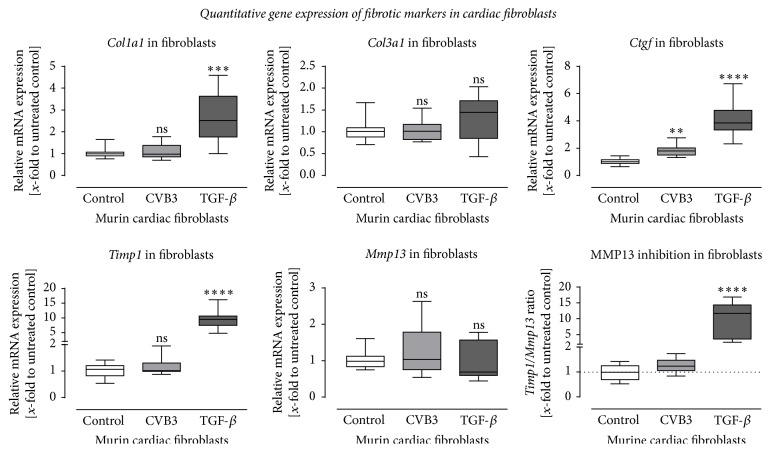
Gene expression analysis of murine cardiac fibroblasts after infection with CVB3 or stimulation with TGF-*β*. Altered gene expression of profibrotic genes was detected in cardiac fibroblasts in response to stimulation with 5 ng/mL TGF-*β* for 24 hours but not after infection with 0.5 MOI CVB3 for 24 hours. The mRNA expression of* Col1a1*,* Ctgf,* and* Timp1* was significantly upregulated after TGF-*β* stimulation but remains unchanged for* Col3a1* and* Mmp13*. Except for a slightly increased expression of* Ctgf* the viral infection with CVB3 did not result in a profibrotic gene expression within 24 hours after infection. Data are presented as relative mRNA expression (*x*-fold to untreated control cells) in box plots using the formula 2^−ΔΔCt^; ^*∗*^significantly different compared to untreated control cells; ^*∗∗*^*P* < 0.01; ^*∗∗∗*^*P* < 0.001; ^*∗∗∗∗*^*P* < 0.0001; ^ns^not  significant.

Gene expression of murine cardiac fibroblasts infected with 0.5 MOI CVB3 was determined, to evaluate similarities between in vivo and in vitro ECM remodeling mechanisms underlying CVB3 infection. In addition, cardiac fibroblasts were stimulated with TGF-*β* for 24 h, mimicking fibroblast activation by immune cells after cardiac inflammation. For transcriptional investigation, mRNA expression levels from* Col1a1*,* Col3a1*,* Ctgf*,* Timp1*, and* Mmp13* were determined (Figure 7). Interestingly, the expression of* Col1a1*,* Ctgf,* and* Timp1* was significantly increased after TGF-*β* treatment (dark-grey bars) compared to untreated controls (white bar), whereas CVB3 infection did not affect the gene expression of* Col1a1*,* Timp1*, and* Mmp13* and showed only a very weak increased expression of* Ctgf*.* Col3a1* and* Mmp13* were not altered by any treatment, compared to the nontreated cells.

## 4. Discussion

In this study, we used the model of CVB3 infection to investigate the long-term effect of viral myocarditis on the myocardium and cardiac function after resolved inflammation. During the acute phase of myocarditis, impaired LV function, severe cardiac inflammation, and fibrosis were observed. The salient findings are that the observed cardiac fibrosis was consistently colocalized with the foci of inflammation, whereas CVB3 infection of cardiac fibroblasts did not induce profibrotic signaling but proinflammatory chemokine expression. Furthermore, after resolved cardiac inflammation, fibrosis was hardly detectable, whereas cardiac function remained impaired. This key finding reveals that cardiac fibrosis seems to be reversible without regaining the hemodynamic function.

### 4.1. Virus Replication and Cardiac Inflammation during Acute Myocarditis

In our previous work, we determined the virus load as well as the cardiac inflammation 3 and 7 days after infection demonstrating higher copy numbers after 7 days [13]. In the here described study, a highly increased viral load was detected in infected hearts 7 days after viral infection. We quantified the (+) RNA and the (−) RNA strand. The (+) RNA strand corresponds to the viral genome of existing or progeny virus particles. The (−) RNA strand is needed as an intermediate to produce progeny viruses. 28 days after infection only very low copy numbers of both RNA strands are still detectable suggesting a nearly complete virus clearance. Interestingly during acute myocarditis, the (+) RNA strand is higher expressed in the myocardium compared to the intermediate (−) RNA strand. Similar results were reported earlier for cardiac tissue demonstrating that the (+) RNA strand is present in a greater excess [7, 23]. Our group reported equal findings in cell culture experiments [30]. Cardiomyocytes as well as cardiac fibroblasts are susceptible for CVB3 infection and in both cell types a ratio between the (+) and the (−) RNA strand of 5 to 10 is described.

As previously shown, cells of the innate immune system (CD68^+^ and CD80^+^) reveal the most prominent signal in clear foci of inflammation [13]. Cells of the innate immune system are the first line of defense for invading pathogens such as virus particles [33]. These cells present proteolytically processed parts of pathogens to cells of the adaptive immune system [34, 35]. These cells are well described to secrete multiple cytokines and growth factors to regulate inflammation [36]. To investigate the cardiac inflammation in more detail, we determined the expression of specific cytokines and chemokines, which were increased during the acute phase of myocarditis. Our group previously showed that recruited monocytes increase the chemokine expression in the infiltrated cardiac tissue in a vicious circle [29].

During the acute phase of CVB3 infection we detected interferon-*β* and interferon-*γ* mRNA expression as induction of antiviral response. Interferon-*β* is mainly synthesized by fibroblasts and known to induce the expression of the anti-inflammatory cytokines IL-10 and TGF-*β* [36]. Experimental and clinical settings have shown that interferon-*β* can lead to an elimination of viral genomes and to an improvement of LV function in patients and animals with enteroviral or adenoviral persistence and LV dysfunction [37–39].

### 4.2. Colocalization of Cardiac Inflammation and Fibrosis during Acute Myocarditis

Moreover, serial cross sections of the cardiac tissue were used to visualize the infiltrated inflammatory cells as well as the cardiac fibrosis. In our histological investigations, the observed cardiac fibrosis was consistently colocalized with the foci of inflammation during the acute phase of myocarditis. Cardiac fibrosis is a biological response to various diseases and can occur as a reparative form of fibrosis during scar formation after myocardial infarction or as a reactive form with increased interstitial collagen content after mechanical overload or inflammation [40, 41].

Furthermore, gene expression analysis of cardiac tissue revealed that collagen-I and the profibrotic* Tgf*-*β* were increased 7 days after infection. As a further profibrotic signal we determined a highly increased gene expression of* Timp1,* the endogenous inhibitor of MMPs, leading to an increased inhibition of matrix degradation and finally fibrosis [42, 43].

### 4.3. Fibroblasts Induce Inflammation: Inflammation Activates Fibroblasts

Fibroblasts are responsible for the production, organization, and turnover of matrix proteins [44]. We here investigated whether viral infection of cardiac fibroblasts itself acts as a profibrotic signal which may explain the developed fibrosis. TGF-*β* stimulation but not viral infection triggered profibrotic signals in infected cardiac fibroblasts.

Until recent, it was suggested that fibroblasts play a relative inert role in the immune system, but in various diseases fibroblast activation leads to production of chemokines and cytokines [45]. For example, in rheumatic arthritis and chronic kidney diseases fibroblasts induce immune cell recruitment to the site of tissue injury [45–47]. Previous work of our group revealed that cardiac fibroblasts, activated by mechanical stress or CVB3, provide proinflammatory mediators inducing the recruitment of inflammatory cells into the cardiac tissue and may therefore act as sentinel cells [29].

**Figure 8:**
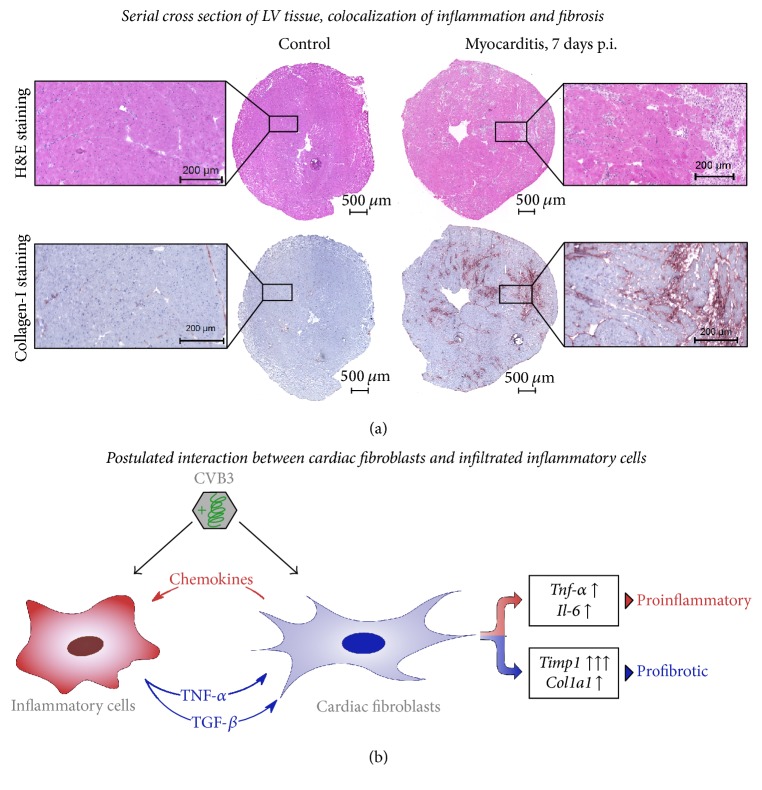
Proinflammatory and profibrotic effects of CVB3 infection in murine heart tissue. (a) Serial cross sections of the left ventricle from healthy or CVB3-infected C57BL/6J mice were stained with hematoxylin/eosin or for COL-I. During acute myocarditis (7 days p.i.) infiltrated cells appear in the cardiac tissue clearly seen as foci of inflammation which are consistently colocalized with fibrosis, shown by COL-I staining. (b) Suggested interaction scheme of cardiac fibroblasts and inflammatory cells in CVB3-induced viral myocarditis. CVB3 infection of cardiac fibroblast alone did not result in profibrotic signaling but increased chemokine and cytokine expression was detected. Elevated chemokine levels might be one regulator to induce the recruitment of inflammatory cells into the infected cardiac tissue. In turn, the induced expression of TGF-*β* in CVB3-infected macrophages might be one key player to induce profibrotic gene expression in cardiac fibroblasts.

Furthermore, activated invading immune cells (e.g., monocytes) display an increased gene expression and an enhanced secretion of TGF-*β*, which then further triggers the transdifferentiation of cardiac fibroblasts into pathologically activated myofibroblasts leading to adverse remodeling [30, 32]. The consistent colocalization of cardiac inflammation and fibrosis (Figure 8(a)) further demonstrated this close interaction between cardiac inflammation and the development of cardiac fibrosis.

These data lead to our postulated interaction scheme illustrated in Figure 8(b). Infected cardiac fibroblasts secrete proinflammatory mediators to recruit inflammatory cells, which then in turn secrete TGF-*β* inducing fibroblast activation leading to cardiac fibrosis. In other fibrosis-related diseased animal models, TGF-*β* antagonism using TGF-*β* neutralizing antibody or TGF-*β* receptor inhibitor led to significantly less cardiac fibrosis [48, 49].

### 4.4. Reversible Cardiac Fibrosis, but Not Restored LV Function

Cardiac dysfunction during myocarditis is caused not only by direct virus-induced myocyte loss [21, 30], but also by inflammatory response of the immune system in order to control the viral infection and cardiac fibrosis [50, 51]. It is already described for the mouse strain C57BL/6J that CVB3 does not induce a chronic ongoing inflammation. Moreover, C57BL/6J mice usually recover from myocarditis [52], which is clearly documented in the resolved inflammation 28 days after infection. Here, we reported increased cardiac fibrosis because of cardiac inflammation during the acute phase of myocarditis. Interestingly, after resolved cardiac inflammation cardiac fibrosis was hardly detectable demonstrating that cardiac fibrosis might be reversible. Gene expression analysis revealed that* Timp1* expression was decreased to basal level resulting in normal MMP activity. We assume that the newly synthesized collagen was not appropriately cross-linked since this postsynthetic collagen processing makes collagen less prone to degradation by collagenases [53]. A regression of cardiac fibrosis is already reported in lisinopril-treated hypertensive patients and hypertensive rats [54, 55].

In tissue from healthy patients, very slow collagen turnover rates have been reported. For example, measuring the racemization of aspartic acid in collagen from human cartilage and skin, a half-life of 117 years for cartilage collagen and 14.8 years for skin collagen was described [56], whereas in rats a collagen turnover time of 30–150 days was described [57].

The hemodynamic characterization clearly displayed a significantly reduced cardiac function of CVB3-infected C57BL/6J mice 7 and 28 days after viral infection. During the acute phase of viral myocarditis (7 days p.i.) systolic and diastolic function were significantly impaired in CVB3-infected mice as an evidence for acute myocarditis accompanied with heart failure symptoms. Interestingly, after 28 days the end-systolic as well as the end-diastolic pressure came up to nearly similar levels compared to control animals, whereas other hemodynamic parameters did not improve. These results suggest a myocardial damage during acute CVB3 infection, which could not be repaired completely to functionally retrieve cardiac function. In cell culture experiments, we have previously shown that CVB3 infects and destroys cardiomyocytes and cardiac fibroblasts [21, 30]. While cardiac fibroblasts can proliferate in the adult myocardium, cardiomyocytes are not able to proliferate and therefore the loss of cardiac muscle cells cannot be compensated to improve the cardiac function [58, 59].

Another reason for impaired cardiac function could be the development of autoimmune myocarditis. It is reported that, especially during the chronic phase of viral myocarditis, mice can develop an autoimmune response to cardiac proteins, such as myosin. This highly depends on the susceptibility of the mouse strain. Fairweather and Rose reported the progression to chronic autoimmune myocarditis following CVB3 infection of susceptible BALB/c mice but not in C57BL/6J mice [60].

## 5. Conclusion

In conclusion, CVB3-infected cardiac fibroblasts induce cardiac inflammation but not fibrosis. Although inflammation is associated with the development of cardiac fibrosis leading to impaired left ventricular function, therefore, cardiac fibroblasts are regulators of cardiac fibrosis as well as cardiac inflammation. Furthermore, the fibrotic process, which comes along with different diseases, is reversible and cardiac fibrosis can decline after resolved inflammation. To investigate the underlying pathophysiological process of reversible fibrosis might be essential to understand the development of heart failure.

## Figures and Tables

**Table 1:** Gravimetric characteristics and hemodynamic function of C57BL/6J mice 7 and 28 days after CVB3 infection.

	Control	CVB3, 7 days p.i.	Change	CVB3, 28 days p.i.	Change
Body weight [g]	29 ± 1	22 ± 1^*∗∗∗*^	−**24%**	24 ± 1^ns^	−**17%**
LV weight [mg]	124 ± 2	80 ± 1^*∗∗∗*^	−**35%**	80 ± 1^*∗∗*^	−**35%**
Global function					
Heart rate [bpm]	585 ± 2	532 ± 8^*∗∗*^	−**9%**	476 ± 12^*∗∗∗∗*^	−**19%**
Cardiac output [mL/min]	15.3 ± 0.9	8.1 ± 0.7^*∗∗*^	−**47%**	6.7 ± 0.6^*∗∗∗*^	−**56%**
Stroke volume [*μ*L]	26.2 ± 1.6	15.2 ± 1.3^*∗∗*^	−**42%**	14.1 ± 1.1^*∗∗*^	−**46%**
Stroke work [*μ*L·mmHg]	2267 ± 172	858 ± 50^*∗∗∗*^	−**62%**	949 ± 118^*∗∗*^	−**58%**
Systolic function					
*P*_es_ [mmHg]	77 ± 3	44 ± 1^*∗∗∗∗*^	−**43%**	64 ± 6^ns^	−**17%**
d*P*/d*t*_max_ [mmHg/s]	9734 ± 407	4080 ± 212^*∗∗∗∗*^	−**58%**	4877 ± 573^*∗∗*^	−**50%**
*V*_es_ [*μ*L]	17 ± 1	20 ± 2^ns^	+**16%**	15 ± 5^ns^	−**12%**
Diastolic function					
*P*_ed_ [mmHg]	4.1 ± 0.3	7.2 ± 0.4^*∗∗∗*^	+**76%**	5.0 ± 0.5^ns^	+**22%**
d*P*/d*t*_min_ [mmHg/s]	−6038 ± 256	−2111 ± 254^*∗∗∗∗*^	−**65%**	−3444 ± 486^*∗*^	−**43%**
Tau [ms]	9.2 ± 0.2	15.3 ± 0.7^*∗∗∗*^	+**65%**	14.5 ± 1.4^*∗∗*^	+**59%**
*V*_ed_ [*μ*L]	42.1 ± 2.1	35.0 ± 1.1^ns^	−**17%**	28.8 ± 5.9^*∗*^	−**32%**

Data are presented as mean ± SEM.

^*∗*^Significantly different compared to noninfected mice (control); ^*∗*^*P* < 0.05; ^*∗∗*^*P* < 0.01; ^*∗∗∗*^*P* < 0.001; ^*∗∗∗∗*^*P* < 0.0001; ^ns^not  significant.

No significances found between CVB3 (7 days) and CVB3 (28 days).

## Additional Points

*Limitation of the Study*. In other fibrosis-related diseased mouse models, TGF-*β* antagonism leads to reduced cardiac fibrosis and finally improves the outcome. Regarding those results, the administration of a neutralizing TGF-*β* antibody during viral myocarditis would prove the key role of TGF-*β* during viral myocarditis but it is not shown in this study.
